# Postoperative abnormal response of C-reactive protein as an indicator for infectious complications after oral oncologic surgery with primary reconstruction

**DOI:** 10.1186/s40463-015-0066-6

**Published:** 2015-04-02

**Authors:** Masaya Akashi, Shungo Furudoi, Kazunobu Hashikawa, Akiko Sakakibara, Takumi Hasegawa, Takashi Shigeta, Tsutomu Minamikawa, Takahide Komori

**Affiliations:** Department of Oral and Maxillofacial Surgery, Kobe University Graduate School of Medicine, Kusunoki-cho 7-5-1, Chuo-ku, Kobe 650-0017 Japan; Department of Plastic Surgery, Kobe University Graduate School of Medicine, Kobe, Japan

**Keywords:** Oral cancer, Infectious complication, Surgical site infection, Non-wound infection, C-reactive protein

## Abstract

**Background:**

C-reactive protein (CRP) screening has been reported to be reliable for detection of infectious complications. Postoperative abnormal response of CRP can predict wound infection in colorectal surgery. This study aimed to determine the efficacy of CRP monitoring to detect infectious complications in oral oncologic surgery.

**Methods:**

One hundred patients who underwent oral cancer resection with primary reconstruction were enrolled. Postoperative kinetics of CRP were classified into a normal or abnormal response.

**Results:**

A normal CRP response after surgery was observed in 61 patients and an abnormal response was observed in 39. There were postoperative infectious complications in 21 patients, with surgical site infections in 13 patients (early onset in six and late onset in seven). Non-wound infections were found in nine patients. Sensitivity, specificity, the positive predictive value, and the negative predictive value for abnormal CRP response as a predictor for early infectious complications were 100%, 70.1%, 35.9%, and 100%, respectively.

**Conclusion:**

Postoperative serial CRP screening is a useful test as an indicator of infectious complications in oral oncologic surgery. Normal CRP responses can rule out almost all early infectious complications.

## Background

Infectious complications after oral oncologic surgery with primary reconstruction are common, and associated with functional morbidity and prolonged hospitalization [1]. Because oral oncologic surgery is a clean-contaminated surgery, the postoperative surgical site infection (SSI) rate is high, occurring in approximately 20% of patients [2,3]. Severe SSI in oral oncologic surgery sometimes causes orocervical fistula, which can be a heavy burden for patients and medical staff. Significant morbidity in the immediate postoperative period is also caused by non-wound infections, with an incidence of 10% in patients who undergo oral cancer surgery, with the majority being pulmonary [4]. Inadequate treatment of pulmonary complications can be life-threatening for patients. Prediction for infectious complications contributes to their appropriate management.

C-reactive protein (CRP) is an acute-phase reactant synthesized by hepatocytes, largely in response to pro-inflammatory cytokines [5]. CRP is not specific for a particular disease because a rise in CRP level is observed with inflammation, trauma, malignancy, and tissue infarction. CRP is present only in trace amounts in healthy subjects, and CRP levels increase within 6 hours after the onset of bacterial infection [6,7]. A rise in CRP levels may be earlier in a pathological process than other non-specific markers (e.g*.*, fever), and falls rapidly on resolution of inflammation [8]. CRP is considered to be useful for detection of an inflammatory response early in its course, and also for monitoring disease activity [5].

A rise in CRP level in acute-phase reactants has been successfully used as a marker of infection after surgical procedures [9,10]. In relation to surgery, the normal CRP response is rapid production of CRP until the peak level is reached, and this postsurgical response (i.e., an initial rise in CRP) is followed by reduction, and an eventual return to the normal range. CRP levels rise postoperatively to a maximum on the 3rd day, and then CRP levels returned to near normal levels on postoperative day 7 [7,11]. These characteristic kinetics of CRP levels after surgery are termed an “increase and decrease pattern”. In eventful cases, “a steady rise or second rise” in CRP level tends to be seen [11]. These abnormal CRP responses are considered to be a predictor for incisional SSI in general surgery such as colorectal surgery, if pneumonia or anastomotic leakage are unlikely or excluded [11].

The purpose of this study was to determine the efficacy of CRP monitoring as a detector of infectious complications in oral oncologic surgery, as well as general surgery.

## Methods

A total of 102 consecutive patients underwent oral cancer resection with primary reconstruction at Kobe University Hospital from May 2009 to October 2013. The criterion for enrollment in this study was 1 month or more of follow-up postoperatively without a loss. Two patients were excluded because of perioperative mortalities, with one patient with postoperative acute respiratory distress syndrome, and one multiorgan failure after surgery. Epidemiological data were retrospectively gathered from the medical charts as follows: age, sex, histological diagnosis, primary tumor sites, TNM classification, diabetes, preoperative radiotherapy, concurrent neck dissection, reconstructive procedures, postoperative infectious complications, including SSI and non-wound infections, estimated blood loss, and surgical time.

All of the patients received prophylactic antibiotic therapy for 3 days after surgery. Blood samples were taken preoperatively, and on postoperative days 1–7. The reference value of CRP was < 4.0 mg/L. The patterns of postoperative CRP kinetics were classified into three groups as follows: (1) CRP values at day 7 that were below 4.0 mg/L without an abnormal response were defined as “early decrease”; (2) an abnormal response included a “second rise” in either parameter at day 5 or 7, with an increase over 3.0 mg/L; and (3) CRP values at day 7 that were over 4.0 mg/L were defined as “delayed decrease”.

The definition of SSI was purulent discharge either spontaneously or by incision, and drainage from the surgical region, including the flap donor site or the presence of an orocutaneous fistula, regardless of etiology within 30 days after surgery [12]. SSI within 14 days after surgery was defined as early onset, and other SSIs were late onset. Perioperative non-wound infections were defined as infections of the tracheobronchial tree, urinary tract, or blood, proven by the isolation of pathogenic organisms from appropriate sources in the clinical settings of fever, sputum, pyuria, or sepsis within 14 days after surgery [4]. Pneumonia was diagnosed with chest-X-ray or computed tomography (CT) suggestive of pneumonia and increased oxygen requirement. Sensitivity, specificity, the positive predictive value (PPV), and the negative predictive value (NPV) were determined based on the results.

Fisher’s exact test was used to identify significant associations among categorical values. Statistical significance was accepted for *p* values of <0.05.

The Institutional Review Board of the Kobe University Hospital approved this retrospective study.

## Results

### Study population

At the time of surgery, the median age of the 100 patients was 68 years (range, 32–90 years), and there were 60 men and 40 women. Histological diagnosis was squamous cell carcinoma in 99 patients and mucoepidermoid carcinoma in one patient. Primary tumor sites were as follows: 35 in the tongue, 24 in the lower gingiva, 15 in the floor of the mouth, 13 in the buccal mucosa, nine in the upper gingiva, three in the mandible, and one in the lower lip. Clinical T-stages were as follows: T2 51, T3 16, and T4 19; N-stages N0 33, N1 24, and N2 29; M-stage M0 86; and recurrence in 14 patients. Twenty-six patients had diabetes. Eight patients underwent preoperative radiotherapy. Unilateral modified radical neck dissection (MRND) was performed in 61 patients, unilateral supraomohyoid neck dissection (SOHND) in 19, bilateral MRND in 11, unilateral MRND/SOHND in one, and neck dissection was not performed in eight. Reconstructive procedures were as follows: radial forearm free flap in 53 patients, rectus abdominis myocutaneous free flap in 25, fibula osteocutaneous free flap in 12, pedicled pectoralis major myocutaneous flap in seven, double free flap (combination of the radial forearm free flap and fibula osteocutaneous free flap) in two, and latissimus dorsi myocutaneous free flap in one. The median operation duration and blood loss were 724.3 minutes (range, 513–1057 minutes) and 891.2 ml (range, 96–4742 ml), respectively.

### Postoperative infectious complications and pattern of CRP response

**Figure 1:**
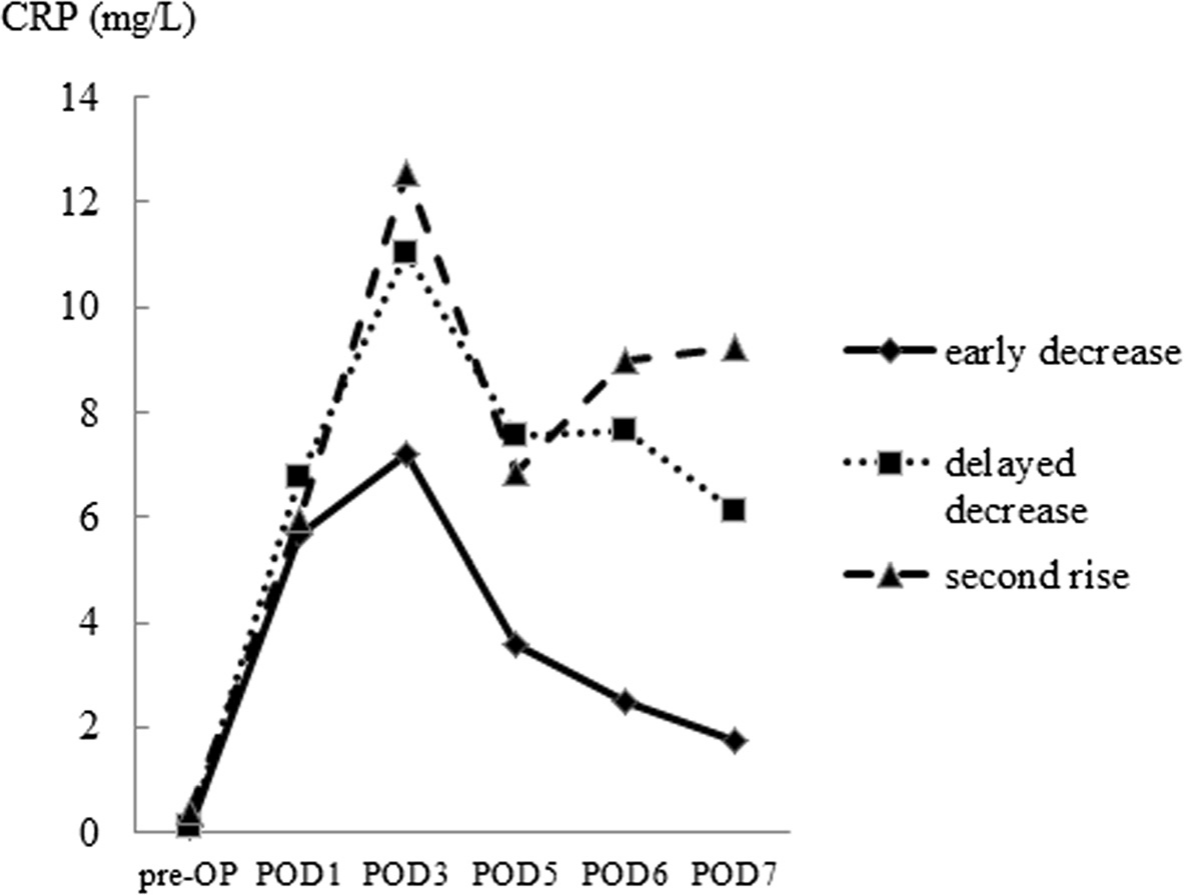
**Patterns of postoperative CRP response.** *Abbreviation*: POD, postoperative day.

After surgery, CRP levels increased in all of the patients and the peak amplitude varied depending on the surgical stress. The median CRP levels in the “early decrease”, “delayed decrease”, and “second rise” groups are shown in Figure 1.

There were postoperative infectious complications in 21 patients. Their characteristics are shown in Table 1. SSIs were found 13 in patients (early onset in six and late onset in seven). Eleven SSIs were in the orocervical region and two were in the donor site of the free fibula flap. Early onset SSIs occurred at a median of 7.5 postoperative days (range, 3–9 days). Late onset SSIs occurred at a median of 26 postoperative days (range, 19–30 days). Non-wound infections were as follows: pneumonia in six patients, catheter-related blood stream infection (CRBSI) in two, and sepsis of unknown cause with positive bacterial blood culture in one. The coincidence of early onset SSI and pneumonia was observed in one patient. Pneumonia, confirmed with chest CT in five patients and with chest X-ray in one patient, was treated with intravenous antibiotic therapy selected by the Division of Infectious Diseases Therapeutics in our hospital (tazobactam/piperacillin [TAZ/PIPC] in two patients, SBT/ABPC in one, TAZ/PIPC and ciproxan in one, and azactam for pseudomonas pneumonia in one). CRBSI was found from an intra-arterial catheter (A-line) in one patient and from a central venous catheter in one patient. Bacteria isolated from blood cultures were Enterobacter species in an A-line case treated with meropenem, and Bacillus species in a central venous catheter case treated with vancomycin. There was no significant association between the occurrence of infectious complications and diabetes (*p* = 0.78) or preoperative radiotherapy (*p* = 0.12).

**Table 1:** **Postoperative infectious complications and CRP response**

**Characteristic**	**No. of Patients (N = 100)**	**Pattern of CRP response (%)**		
		**Early decrease**	**Delayed decrease**	**Second rise**
		**(N = 61)**	**(N = 31)**	**(N = 8)**
Infectious complications	21	4 (6.6)	11 (35.5)	6 (75.0)
*SSI*	13	4 (6.6)	9 (29.0)	–
Early onset	6	–	6 (19.4)	–
Late onset	7	4 (6.6)	3 (9.7)	–
*Non-wound infections*	9	–	3 (9.7)	6 (75.0)
Pneumonia	6	–	2 (6.5)	4 (50.0)
CRBSI	2	–	1 (3.7)	1 (12.5)
Unknown cause	1	–	–	1 (12.5)

*Abbreviations*: *SSI* surgical site infections, *CRBSI* catheter related blood stream infection.

Six of eight patients with a second rise in CRP (75.0%) had postoperative infectious complications (pneumonia, four; CRBSI, one; sepsis of unknown cause, one). Eleven of 31 patients with a delayed decrease in CRP (35.5%) had postoperative infectious complications (early onset SSI, five; late onset SSI, three; pneumonia, one; CRBSI, one; and coincidence of early onset SSI and pneumonia, one). Four of 61 patients with an early decrease in CRP (6.6%) had late onset SSIs (Table 1).

When a positive test was defined as a postoperative abnormal response of CRP, including a delayed decrease and second rise, examination of CRP for predicting postoperative infectious complications showed true positive results in 17 and false positive results in 22 patients. True negative results were recorded in 57 patients and four false negative results were observed. Except for cases of late onset SSI, CRP monitoring for early infectious complications showed true positive results in 14 patients and false positive results in 25 patients. True negative results were recorded in 61 patients, and no false negative result was observed. Sensitivity, specificity, PPV, and NPV for all postoperative infectious complications, as well as those, except for late onset SSI, are shown in Table 2.

**Table 2:** **Prediction of infectious complications**

**Infectious complications**	**Sensitivity**	**Specificity**	**PPV**	**NPV%**
All	80.1	72.2	43.6	93.4
Except for late onset SSI	100	70.1	35.9	100

*Abbreviations*: SSI, surgical site infections; PPV, positive predictive value; NPV, negative predictive value.

## Discussion

The postoperative course of primary reconstruction for large defects following oral cancer resection has complex and diverse complications. SSI often requires long-term wound treatment and prolonged hospital stays, which are a heavy burden for patients and medical staff. Although the incidence of postoperative wound infections after head and neck cancer surgery without administration of perioperative antibiotics ranges from 24–87% [13], some prospective studies recently reported that the wound infection rate has decreased in the range of 14–40% through prophylactic antibiotics [4,14]. The occurrence rate of SSI, particularly after oral cancer surgery, was reported as 19.8% by Liu et al. [2] and 21% by Cloke et al. [3] Postoperative pulmonary complications are also common (10–47%) in head and neck reconstructive surgery, and lead to longer hospital stays and increase mortality [15-20]. In our study, the occurrence rate of postoperative infectious complications was 21% (SSI, 13%; non-wound infection, 9%) under the administration of prophylaxis antibiotics.

Comprehension of the postoperative course in eventful cases and reliable screening tests for prediction of postoperative infections would contribute to appropriate treatment of postoperative infectious complications and improve postsurgical outcome. In some reports, postoperative CRP monitoring with a focus on abnormal CRP response (i.e*.*, a steady or second rise in CRP from postoperative days 5 to 7) was considered to be a reliable predictor of SSI [7,11]. Kang et al. reported that the sensitivity, specificity, PPV, and NPV for an abnormal CRP response were 100%, 96.8%, 31.3%, and 100%, respectively, as a predictor for early onset SSI after spinal surgery [7]. Based on the time to clinical presentation, SSI can be categorized as early, delayed, or late onset. CRP monitoring in the early period after surgery might have a limitation in detecting late onset SSI, which might have a hematogenous origin or result from the intraoperative seeding of microbes, with infections remaining subclinical for an extended period [7]. All four false negative cases in this study were late onset SSI due to an infected old hematoma. This finding indicates that late onset SSI is difficult to predict only by perioperative CRP monitoring, as previously shown. Fujii et al. [11] reported that persistent elevation of CRP was predictive of incisional SSI (sensitivity, 71.4%; specificity, 83.1%) in colorectal surgery if pneumonia or anastomotic leakage was unlikely or excluded. Another report showed that a second rise or failure to achieve a decrease in postoperative CRP values had good sensitivity and a good predictive value for postoperative infections [21]. We defined a deviation from normal CRP kinetics after surgery as a second rise or delayed decrease. In the second rise group, all infectious complications were non-wound infection, while a delayed decrease in CRP was observed in early onset SSI cases. The sensitivity, specificity, PPV, and NPV for early infectious complications in our study were similar to those previously reported [7]. However, there are differences between oral oncologic surgery and spinal or colorectal surgery as follows. (1) Oral oncologic surgery has traditionally been considered clean-contaminated surgery. (2) Postoperative pulmonary complications are common among patients undergoing oral oncologic surgery with primary reconstruction requiring tracheostomy and planned postoperative mechanical ventilation in an intensive care unit. (3) There is a difference in surgical invasiveness between oral oncologic surgery and spinal or colorectal surgery.

Early identification of infections is still a challenge for clinicians. A previous meta-analysis reported that procalcitonin levels are more accurate markers for bacterial infections than CRP levels [22], while the usefulness of perioperative CRP monitoring in general surgery is generally accepted. Although Cole et al. [5] found no correlation between CRP levels and the occurrence of infection within the first 3 days after surgery, other studies have confirmed that CRP is not a good indicator of the presence of early postoperative infection [23]. We consider that a rise in CRP level after postoperative day 3 may indicate infection. Therefore, CRP has a clear role in monitoring response to treatment when infection is diagnosed [24]. Kang et al. mentioned that their management strategy for a postoperative abnormal CRP response, including a steady and second rise in CRP levels at postoperative day 5 or 7, was an immediate return to antibiotic therapy with a different regimen [7]. This strategy was decided upon because an abnormal CRP rise suggests that prophylactic antibiotics may be ineffective. When there is a postoperative abnormal CRP response with no clear evidence of intercurrent infection or other inflammatory processes, close observation for signs of SSI and serial monitoring of laboratory parameters are considered to be important [7].

In CRP “second rise” group in this study, all infectious complications were non-wound infections, treated with intravenous antibiotic therapies selected by the Division of Infectious Diseases Therapeutics in our hospital. CRP “delayed decrease” group in this study could be further classified into two patterns of kinetics as follows. (A) The maximum peak level of CRP on the 3rd day after surgery was so high with a subsequent steady decline, and CRP levels on postoperative day 7 could not recover within the normal range. (B) CRP levels within the first 3 days were not so high without a subsequent decrease between postoperative day 5 and 7. The former might not require prolonging or changing antibiotics in uncomplicated cases, irrespective of a high level of CRP. The latter should be kept under careful observation for signs of infection.

In conclusion, postoperative serial CRP screening is considered as one of the reliable indicators of postoperative infectious complications in oral oncologic surgery. A normal CRP response can rule out almost all postoperative infectious complications, except for late onset SSI, which is often caused by hematogenous origin. Therefore, late onset SSI can probably be prevented by intraoperative hemostasis and appropriate drainage.
